# High versus low pneumoperitoneum PressUre for parenchymal transection in minimally invasive major liver surgery (PPULS)—a non-inferiority, multicenter, randomized, controlled trial

**DOI:** 10.1186/s13063-025-09269-9

**Published:** 2025-12-01

**Authors:** Esther Giehl-Brown, Elias Khajeh, Sarah Dehne, Zoltan Czigany, Oliver Gutzeit, Christopher Neuhaus, Carina Riediger, Emrullah Birgin, Nuh Rahbari, Mathieu D’Hondt, Georg Lurje, Markus Weigand, Christoph Michalski, Arianeb Mehrabi, Christoph Kahlert

**Affiliations:** 1 Department of General, Visceral and Transplant Surgery, Heidelberg University Hospital, Im Neuenheimer Feld 420, Heidelberg, 69120 Germany; 2 Department of Anesthesiology, Heidelberg University Hospital, Heidelberg, Germany; 3 Department of Visceral, Thoracic and Vascular Surgery, Faculty of Medicine and University Hospital Carl Gustav Carus, TU Dresden, Dresden, Germany; 4 Department of General and Visceral Surgery, University Hospital Ulm, Ulm, Germany; 5 Department of Digestive and Hepatobiliary/Pancreatic Surgery, Groeninge Hospital, Kortrijk, Belgium

**Keywords:** Pneumoperitoneum, Minimally invasive surgery, Parenchymal dissection, Liver surgery, Intraoperative blood loss, CO_2_ embolism

## Abstract

**Background:**

Low pneumoperitoneum pressure (LPP) lowers the incidence of CO_2_ embolisms in minimally invasive liver resections (MILR), while higher pneumoperitoneum pressure (HPP) reduces intraoperative blood loss. This contradiction necessitates careful pressure management especially in major liver resections where intraoperative blood loss greatly impacts postoperative outcome.

**Methods:**

In this randomized non-inferiority trial, adults undergoing elective MILR for any indication will be recruited in alignment with inclusion and exclusion criteria. After given informed consent, eligible patients will be randomized to either low (≤10 mmHg) or high (≥14 mmHg) pneumoperitoneum pressure during parenchymal transection. Blood, peritoneal biopsies, and liver tissue will be sampled to evaluate intraoperative tissue damage. Sample size (*n* = 66 patients per group) is calculated based on the current literature. The primary study endpoint is intraoperative blood loss during the parenchymal transection phase. Secondary endpoints include CO_2_ embolisms, intraoperative tissue damage, operation time, morbidity, mortality, and duration of hospitalization.

**Discussion:**

Minimizing intraoperative blood loss in MILR is a clinically relevant problem, which greatly impacts the procedure’s safety and influences the patient’s morbidity and mortality. HPP, exerting counter pressure to the vascular pressure, serves for bleeding control in MILR. The risk of CO_2_ embolism, arising from the combination of high intra-abdominal pressure and low central venous pressure, favors the use of LPP. The proposed trial aims to assess the non-inferiority of LPP compared to HPP during the parenchymal transection phase of MILR.

**Trial registration:**

ClinicalTrials.gov NCT06770803. First Submitted: 2024-12-30, First Submitted that Met QC Criteria: 2025-01-07, First Posted: 2025-01-13.

**Supplementary Information:**

The online version contains supplementary material available at 10.1186/s13063-025-09269-9.

## Introduction

### Background and rationale {6a}

Minimally invasive techniques in liver surgery gain popularity as they facilitate postoperative recovery while achieving comparable oncologic outcomes to the open approach [1]. Since the first worldwide approval of the da Vinci® robotics platform in 2001, da Vinci®-assisted liver surgery has even further enhanced the application of laparoscopic surgery by implementing a three-dimensional view, the magnification of the operative field, and the seven degrees of freedom of the human hand, thereby overcoming the limitations of conventional laparoscopic surgery [2, 3]. No consensus on the application of pneumoperitoneum pressure in minimally invasive liver resections (MILR) has been reached yet, as prospective clinical studies are scarce. All minimally invasive techniques require carbon dioxide (CO_2_) for the induction and maintenance of the pneumoperitoneum, which can lead to the formation of CO_2_ embolisms potentially causing pulmonary edema, cerebral infarction, shock, and cardiac arrest [4]. In contrast, the positive pressure of the CO_2_ pneumoperitoneum reduces intraoperative blood loss during MILR alongside the development of new transection devices and advancements in inflow control [5, 6]. Intraoperative blood loss greatly impacts postoperative survival following liver surgery [7], and perioperative transfusions of packed red blood cells in oncologic liver resections represent an independent poor prognostic indicator for overall survival [8]. Low-pressure pneumoperitoneum on the other hand has been shown to decrease postoperative pain scores and analgesic consumption in comparison to standard pneumoperitoneum [9], and international guidelines recommend the application of “the lowest intra-abdominal pressure allowing adequate exposure of the operative field rather than a routine pressure” [10]. Nevertheless, evidence for the application of low-pressure pneumoperitoneum is only moderate to low, requiring additional studies to better define its safety [9]. MILRs are associated with an elevated risk of CO_2_ embolism due to the risk of rupture of the hepatic veins or exposure of the hepatic sinusoids. Luo et al. demonstrated in a recently published randomized controlled trial that a pneumoperitoneal pressure of 10 mmHg reduced the incidence and duration of severe CO_2_ embolisms during laparoscopic liver resection in comparison to standard 15 mmHg [11]. Additionally, low-pressure pneumoperitoneum was particularly beneficial to the subgroup of liver resections that were adjacent to the second hepatic hilum [11]. These results reinforce the correlation of the pneumoperitoneum pressure and the risk for CO_2_ embolisms. An additional advantage of low-pressure peritoneum is improved postoperative recovery [12]. No significant difference in intraoperative blood loss or the amount of blood transfusions was observed in the study by Luo et al., but 93.6% of all participants underwent minor and only 6.4% major resections [11]. An experimental study on piglets showed that high intra-abdominal pressure (16 mmHg) reduced bleeding in comparison to low pressure (8 mmHg) [13]. Other experimental, retrospective, and prospective comparative studies further strengthen the hypothesis that high pneumoperitoneum reduces blood loss [14].

To address this contradiction, we propose a randomized non-inferiority trial to compare low-pressure to high-pressure pneumoperitoneum during the transection phase of major MILR and its effect on intraoperative blood loss and the occurrence of embolic complications.

### Objectives {7}

The objective of this trial is to determine whether the maintenance of a low intraperitoneal insufflation pressure (IIP) of ≤10 mmHg during the parenchymal transection phase of conventional and robotic-assisted laparoscopic liver resections is non-inferior to a higher IIP of ≥14 mmHg in terms of intraoperative blood loss, gas embolisms, perioperative morbidity, and mortality.

The primary research question is as follows: Is the maintenance of a low IIP of ≤10 mmHg during the parenchymal transection phase of conventional or robotic-assisted laparoscopic liver resections non-inferior to a higher IIP of ≥14 mmHg in terms of intraoperative blood loss during the parenchymal transection phase?

### Trial design {8}

**Fig. 1:**
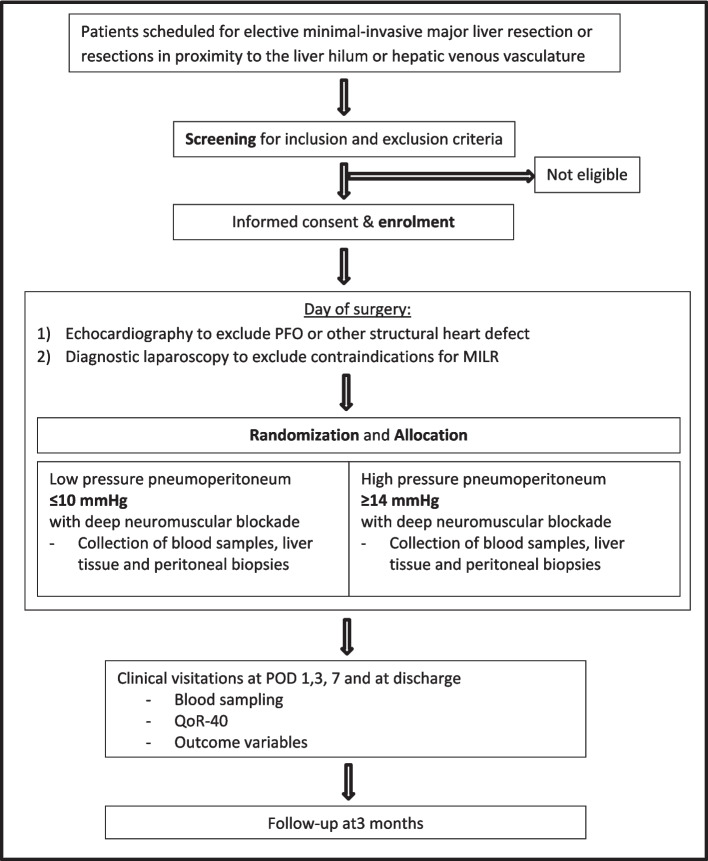
CONSORT flowchart depicting the screening, enrollment, randomization, intervention, and follow-up assessments

This study is a two-arm parallel-group, randomized non-inferiority trial in patients undergoing elective minimally invasive liver surgery with an allocation ratio of 1:1 per hospital and a follow-up period of 3 months. The flow diagram of the study is presented in Fig. 1.

## Methods: participants, interventions, and outcomes

### Study settings {9}

All patients being scheduled for elective conventional or da Vinci robotic-assisted laparoscopic liver resections for any benign or malignant indication will be screened for inclusion at the Department of General, Visceral and Transplant Surgery at Heidelberg University Hospital or collaborating hospitals.

### Eligibility criteria {10}

Inclusion criteria consist of:

1. Age equal or older than 18 years.
2. Capacity of consent.
3. Planned elective conventional laparoscopic or da Vinci-assisted major liver resection or resections near the liver hilum or the hepatic venous vasculature. Major liver resections are defined as the resection of 3 liver segments or more (right and left partial hepatectomies, extended right and left hepatectomies, liver resections of 3 or more segments). Right posterior sectionectomies and mesohepatectomies of ≥2 liver segments are considered resections in proximity to the liver hilum or hepatic venous vasculature.

All study participants must give informed consent before study enrollment.

Exclusion criteria represent:

1. The participation in another trial with interference of intervention of this study
2. Being a woman who is pregnant or breast-feeding or planning to become pregnant
3. American Society of Anesthesiologists (AS) score >3
4. Language barrier
5. Any contraindication to a minimally invasive surgical approach or intolerance to pneumoperitoneum
6. A patent foramen ovale (PFO) or any other structural cardiac defect that facilitates paradoxical gas embolisms
7. Diagnosis of neuromuscular disease, heart failure NYHA > class II, or chronic obstructive pulmonary disease (COPD)
8. Being on oral anticoagulation therapy other than aspirin 100 mg daily or any other condition known to increase the risk of bleeding

### Who will take informed consent? {26a}

A qualified physician, trained in the study protocol and consent procedures, will obtain informed consent. The physician will explain the study’s purpose and interventions, its potential risks and benefits, and the voluntary nature of participation. Written informed consent will be secured before any study procedures begin.

A model of the written informed consent form used in this study is provided as Supplementary File 1.

### Additional consent provisions for collection and use of participant data and biological specimens {26b}

The main informed consent form includes comprehensive information regarding the collection, storage, and future use of participant data and biological specimens. The form clearly outlines the types of data and specimens that will be collected, details the procedures for anonymization and secure storage, and describes any potential future research uses. By signing the form, participants agree to these terms as part of their overall study participation.

## Interventions

### Explanation for the choice of comparators {6b}

Evidence for the application of low-pressure pneumoperitoneum is only moderate to low. Therefore, we propose a randomized non-inferiority trial to investigate the effect of low-pressure in comparison to high-pressure pneumoperitoneum during the transection phase of major MILR on intraoperative blood loss during this phase while also evaluating the risk of CO_2_ embolisms.

### Intervention description {11a}

Intraperitoneal insufflation pressures (IIP) during the parenchymal transection phase of liver resection will be different between the two study groups. Baseline IIP of the surgical procedure will be ≤10 mmHg. IIP will be elevated to ≥14 mmHg in the intervention group during the parenchymal transection phase of liver resection, while IIP will be maintained at ≤10 mmHg in the control group. Deep neuromuscular blockade will be applied in all patients and is defined as a train-of-four (TOF) of 0 or posttetanic count of 1–2. The quality of the surgical field is rated every 8 min on the Leiden Surgical Rating Scale (L-SRS) during the transection phase and every 15 min outside of it. When the field conditions reach inadequate scores of ≤3, IAP will be increased accordingly in steps of 2 mmHg.

Pringle maneuver, the clamping of the hepatoduodenal ligament, is obligatory and predefined as a sequence of 20 min of clamping followed by 5 min of perfusion.

### Criteria for discontinuing or modifying allocated interventions {11b}

Individual withdrawal criteria will include the participant’s right to withdraw consent at any time without any negative consequences for their subsequent medical care. Participants may choose to withdraw if they experience adverse events or if they feel uncomfortable continuing in the study for any reason. Additionally, any significant changes in their medical condition that may affect their safety or the validity of the trial, such as newly diagnosed medical conditions or complications related to the surgical procedure, may also warrant withdrawal from the study. The research team will ensure that participants are fully informed of their right to discontinue their participation at any point during the trial, and efforts will be made to document the reasons for withdrawal if disclosed.

Withdrawal criteria for the overall PPULS trial may arise from significant changes in the safety landscape during the study period, such as emerging data that indicate an unacceptable level of risk for participants. This could include new findings related to the incidence of adverse events associated with the pneumoperitoneum pressures being tested, or if there are changes in clinical guidelines that recommend against the studied interventions. This trial will be discontinued only for reasons pertaining to efficacy, safety, or feasibility. However, at this stage, no specific overall withdrawal criteria have been identified; the study is designed with appropriate monitoring and safety protocols in place to ensure participant welfare throughout its duration.

### Strategies to improve adherence to interventions {11c}

Not applicable as the intervention is performed during surgery.

### Relevant concomitant care permitted or prohibited during the trial {11d}

Not applicable as patients receive the best medical care independent of their study participation.

### Provisions for post-trial care {30}

Not applicable as no provisions are available through this trial.

### Outcomes {12}

#### Primary outcome

The primary study endpoint is intraoperative blood loss in milliliters measured during the parenchymal transection phase. It will be referred to as *intraoperative blood loss during the parenchymal transection phase*.

#### Secondary outcomes

The secondary endpoints of the study are defined as follows:

- Intraoperative blood loss in milliliters measured during the overall surgical procedure (from first skin incision to full skin closure)
- Incidence of CO_2_ embolisms in the right atrioventricular system detected by intraoperative transesophageal echocardiography with a mid-esophageal right ventricular inflow-outflow (RVOT) view before, during, and after the parenchymal transection phase. The occurrence of CO_2_ embolisms will be graded according to the “Tübingen Venous Air Embolism Grading Scale” [15]
- Other surgery-related secondary endpoints include:
  1. The quality of the surgical field (L-SRS)
  2. The overall time of surgery and the duration of the parenchymal transection phase in minutes
  3. IIP at the end of the parenchymal transection phase and mean IIP outside of the parenchymal transection phase, number of surgeries with an increase of +2 mmHg, +4 mmHg, or +6 mmHg, etc.
  4. Occurrence of intraoperative surgical complications other than gas embolism
  5. Conversion rate to open laparotomy
  6. Difficulty of liver resection according to the “scoring system for laparoscopic liver resection”
  7. Indocyanine green (ICG) clearance after the parenchymal transection phase and 24 h later
- Other anesthesia-related secondary outcomes include:
  1. Application of perioperative peridural catheter (drug, dosage, duration)
  2. Mean dose of narcotic drugs (propofol, sufentanil, remifentanil, lidocaine, esketamine, rocuronium, cisatracurium, and others)
  3. Transfusion rate and coagulation factor administration
  4. Neuromuscular blockade reversal (sugammadex and others)
  5. Usage of vasopressors (epinephrine, noradrenaline, dobutamine, theodrenaline-cafedrine, and others)
  6. Use of buffer solutions (natrium bicarbonate and tris(hydroxymethyl)aminomethane)
  7. Mean EtCO_2_ and occurrence of an abrupt end-tidal carbon dioxide (EtCO_2_) partial pressure decrease ≥5 mmHg and mean arterial carbon dioxide pressure (paCO_2_), pH and bicarbonate at 15-min intervals during the parenchymal transection phase and every 30 min outside of it
  8. A sudden decrease in MAP > 20 mmHg
  9. Abnormal regional cerebral oxygen saturation (rScO_2_) (defined as a decrease in rScO_2_ > 15% compared with baseline or rScO_2_ < 50%)
  10. Mean central venous pressure (CVP) and increased CVP (CVP > 10) at 15-min intervals
  11. Peripheral oxygen saturation (SpO_2_) <90%, or changes in SpO_2_ or hypoxia (SpO_2_ < 90% lasting at least 1 min)
  12. Heart rate (HR)
  13. Mean arterial pressure (MAP) and hypotension (MAP < 65 mmHg)
  14. Arterial lactate
  15. Ventilation parameters: mean positive end expiratory pressure (PEEP), inspiratory pressure (Pinsp), mean airway pressure (Pmean), breathing rate
  16. Perioperative fluid balance
- Postoperative secondary outcomes include:
  1. Surgery-related postoperative complications (e.g., wound impairments, biliary leakage, postoperative liver failure, pulmonary complications)
  2. Overall postoperative morbidity within 30 postoperative days measured via the Comprehensive Complication Index (CCI) [16] according to the Clavien-Dindo classification [17]
  3. Dose of postoperative opioid analgesics (piritramide and others) and dose of postoperative non-opioid analgesics (metamizole, paracetamol, and others) for 48 h after the surgery
  4. Postoperative laboratory values (e.g., bilirubin, c-reactive protein (CRP), international normalized ratio (INR))
  5. Length of the intensive care unit (ICU) stays, readmissions to the ICU, and overall hospital stay
  6. Nausea and vomiting, use of emetic medications, day of first postoperative bowel movement, use of prokinetic medications
  7. Quality of recovery based on the Quality of Recovery 40 (QoR-40) questionnaire on postoperative days 1, 3, and 7
  8. Histopathology outcomes (e.g., lymph node yield, perineural and lymphovascular invasion)
  9. Postoperative neurological recovery according to the National Institutes of Health Stroke Scale (NIHSS)
- Outcomes assessed during follow-up include:
  1. 30-day and 90-day survival.
  2. Hospital readmission within 30 days of remission.
  3. In case of a malignant diagnosis, oncologic outcome (overall survival and disease-free survival) will be evaluated based on [18] surgical margins (R0—no evident tumor along the transection surface or within 1 mm of it; R1—microscopically positive; R2—grossly positive).
- To assess the effect of pneumoperitoneum pressure on tissue damage:
  1. Plasma damage-associated molecular patterns (DAMPs), for example, heat shock protein 70 (HSP70), high mobility group box 1 (HMGB1), nuclear deoxyribonucleic acid (nDNA), and mitochondrial DNA (mtDNA).
  2. Plasma cytokines (for example, tumor necrosis factor alpha (TNFα), interleukin-10 (IL-10), and IL-6) will be evaluated from blood samples drawn directly before the parenchymal phase and at the end of it, as well as on postoperative days 1 and 3.
    In addition, liver and peritoneal biopsies will be taken before and after the parenchymal transection phase of the liver resection. Biopsies will be used to extract mRNA and determine:
  3. Levels of hypoxia-inducible factor 1-alpha (HIF1α), TNFα, IL-1β, IL-6, vascular endothelial growth factor (VEGF), hepatocyte growth factor (HGF), and platelet-derived growth factor (PDGF).

### Participant timeline {13}

**Fig. 2:**
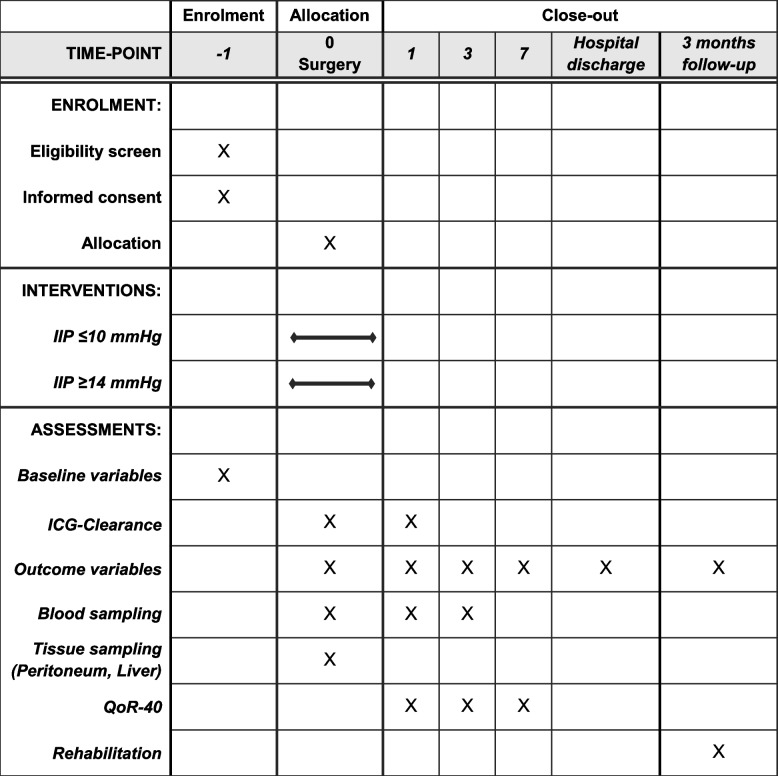
The following SPIRIT figure outlines the schedule of enrolment, interventions, and assessments

The following SPIRIT figure outlines the schedule of all study timepoints [19] (Fig. 2).

### Sample size {14}

The sample size calculation is based on the primary endpoint “intraoperative blood loss during the parenchymal transection phase.” A difference of 100 ml in blood loss is considered clinically relevant, setting the non-inferiority margin at 100. Assuming no difference in mean blood loss between the two groups and using a standard deviation of 184 for blood loss in minimally invasive major liver resection [20], the initial single-center sample size required to achieve 80% power (*β* = 0.2) at *α* = 0.05 was calculated as 42 per group. Adjusting for a multicenter study design with an intra-class correlation coefficient (ICC) of 0.05 across four centers, the required sample size increases to 66 patients per group. Accounting for a 5% loss to follow-up, we plan to include a total of 132 patients across both study arms.

### Recruitment {15}

Recruitment of participants will be performed at the Heidelberg University Hospital, Ulm University Hospital, and the University Hospital Carl Gustav Carus Dresden, Germany, and at the Groeninge Hospital Kortrijk, Belgium.

## Assignment of interventions: allocation

### Sequence generation {16a}

The patients are intraoperatively randomized to either the control arm (low intra-abdominal pressure during parenchymal dissection) or the intervention arm (high intra-abdominal pressure), each in combination with deep neuromuscular blockade.

Prior to randomization, transesophageal echocardiography is performed to exclude a patent foramen ovale (PFO) or other structural cardiac defects associated with an increased risk of paradoxical gas embolism. Diagnostic laparoscopy is performed to rule out intra-abdominal adhesions, peritoneal carcinomatosis, or other contraindications to a minimally invasive approach or liver resection.

#### Stratification variables

Randomization is stratified according to the following variables:

- Center: to account for potential differences among participating clinics
- Surgical procedure: conventional laparoscopic versus robot-assisted

### Concealment mechanism {16b}

Randomization is conducted as stratified block randomization with a 1:1 allocation ratio. Block sizes remain confidential until the end of recruitment. The REDCap Randomization Module Software is used to execute the randomization process. Allocation and assignment of participants will be conducted by members of the research team who do not have access to participants’ information other than the study identification code, the center, and the planned surgical procedure.

### Implementation {16c}

A qualified study physician or nurse will generate the allocation sequence, enroll participants, and assign them to interventions.

## Assignment of interventions: blinding

### Who will be blinded [21]

Due to the nature of the intervention, all staff members in the operating theater cannot be blinded. Therefore, blinding to group allocation will be limited. In both groups, the surgeons and assessors of outcomes are blinded to the study arm and intra-abdominal pressure. The anesthesiologists, who are responsible for the possible treatment of CO_2_ embolism, are not blinded to treatment allocation. The project coordinators, data managers, and the intervention coordinator will have access to group assignment but will not be involved in assessing participants in the clinical visits and follow-up. Baseline visit assessments will be conducted prior to randomization.

### Procedure for unblinding if needed [21]

Unblinding is allowed on the occasion of the occurrence of severe gas embolism.

## Data collection and management

### Plans for assessment and collection of outcomes {18a}

An outline of the study visits and a description of assessments are shown in Table 1.

**Table 1:** Description of the time points and assessments of the study visits

Visit	Day	Description of assessments
Phase I: screening and inclusion in the study (intervention and control groups)		
V1	Outpatient clinic	- Inclusion/exclusion criteria - Informed consent - Assessment of sociodemographic data and medical history (*CRF 1*)
Phase II: intervention		
V2	Day of surgery	- Randomization to intervention/control group - Assessment of the surgical procedure (*CRF 2*) - Assessment of the anesthesia (*CRF 2*) - Collection of blood samples and peritoneal tissue samples at the beginning and the end of the parenchymal transection phase - Sampling of liver tissue from the liver resection specimen
Phase III: rehabilitation		
V3	POD 1	- Assessment of QoR-40 - Blood sampling
V4	POD 3	- Assessment of QoR-40 - Blood sampling
V5	POD 7	- Assessment of QoR-40
V6	Discharge from hospital	- Assessment of postoperative recovery (*CRF 3*)
Phase IV: follow-up		
V7	Follow-up at 3 months	- Telephone interview to assess outpatient rehabilitation (*CRF 4*)

### Plans to promote participant retention and complete follow-up {18b}

Participant retention is promoted by integrating the study follow-up into routine clinical care. Patients are often scheduled for a standard follow-up at 3 months following surgery. No additional visits are required; if an in-person follow-up is not feasible, a phone call will be conducted to ensure complete data collection.

### Data management {19}

The names of the patients/participants and all other confidential information are subject to medical confidentiality and the provisions of the General Data Protection Regulation (GDPR) as well as the relevant state or federal data protection laws (LDSG or BDSG). Data will be stored for a maximum of 10 years following the conclusion of the study. Only the principal investigator will possess the pseudonymization code, and the anonymization of personal data will be carried out in accordance with § 35 Abs. 2 LDSG BW once the data is integrated into the database. Disclosure of patient/participant data will only occur, if necessary, in a specific format. Third parties will not have access to original documents.

### Confidentiality {27}

Medical confidentiality and data protection regulations will be strictly observed. During the study, medical findings and personal information about the participants will be collected and recorded either in a personal file at the study site or electronically stored. Data relevant to the study will additionally be stored in a pseudonymized form. The study management will take all reasonable measures to protect the data in accordance with the European Union’s data protection standards. The data will be secured against unauthorized access, and decoding will occur only in case of withdrawal from the study and data destruction is required.

### Plans for collection, laboratory evaluation, and storage of biological specimens for genetic or molecular analysis in this trial {33}

As part of the PPULS study, biological specimens will be collected to evaluate the effects of different intra-abdominal pressures during surgery. During the procedure, two blood samples and two tissue biopsies (from the liver and peritoneum) will be collected to investigate the impact of varying pressures on tissue integrity. Following surgery, additional blood samples (approximately 15 ml per visit, totaling 45 ml) will be taken on days 1, 3, and 7 to monitor inflammatory markers and tissue damage. The collected specimens will be securely stored and used for molecular analysis within this study. After project termination, samples and data will be safely stored for 10 years and then disposed of. Their future use in ancillary studies is not yet planned.

## Statistical methods

### Statistical methods for primary and secondary outcomes {20a}

The data will be managed and analyzed according to the applicable standard operating procedures (SOPs) within the shared unit of the clinical trial center. All relevant study information will be documented in case report forms (CRFs), which will be reviewed by the study team prior to data entry. A safety analysis will be conducted for all patients who underwent one of the interventions. Serious adverse events will be documented immediately, prompting notification of the principal investigator. Absolute and relative frequencies, as well as the severity and relationship to the intervention, will be compared between intervention groups.

The modified intention-to-treat (mITT) analysis will be used to evaluate the primary and secondary endpoints. The mITT set includes all randomized patients who initiated the intervention and met intraoperative inclusion criteria (e.g., exclusion of a PFO). Patients excluded intraoperatively due to contraindications (e.g., adhesions or peritoneal carcinomatosis) will not be included in the analysis.

The primary endpoint (intraoperative blood loss during the parenchymal transection phase) will be analyzed using linear regression if data follow a normal distribution. If normality is not met, a non-parametric bootstrap method will be employed to calculate confidence intervals. The 90% confidence interval (CI) for the mean difference between the two groups will be calculated, and the lower bound of this interval will be compared to the predefined non-inferiority margin of 100 ml to confirm non-inferiority. The corresponding *p* value will also be computed.

Secondary endpoints will also be analyzed:

- Continuous variables: These will be analyzed using descriptive statistics (mean, standard deviation, quartiles) along with two-sided 95% confidence intervals (CIs).
- Categorical variables: Absolute and relative frequencies will be reported, and two-sided *p* values will be calculated using the chi-squared test or Fisher’s exact test.

All statistical tests will be two-sided and conducted at a significance level of *α* = 0.05. Analyses will account for stratification to minimize bias and ensure accurate *p* values [22].

### Interim analyses {21b}

Adverse events and clinically significant deterioration will be monitored, discussed, and reported. The lead statisticians will look for clinically significant deterioration on any outcome at the midpoint of the trials. Clinically significant deterioration will be determined through RCI analyses from baseline to the immediate post-treatment. If clinically significant deterioration is detected, a standardized procedure will follow.

### Methods for additional analyses (e.g., subgroup analyses) {20b}

Not applicable as no subgroup analyses are planned.

### Methods in analysis to handle protocol non-adherence and any statistical methods to handle missing data {20c}

For the primary analysis, a complete-case analysis will be performed, considering only patients with complete data. Missing data will not be imputed unless the proportion of missing data is determined to be significant. Additionally, a multiple imputation method will be employed as a sensitivity analysis to assess the robustness of the results in the presence of missing data and to minimize potential bias from non-randomly missing values.

### Plans to give access to the full protocol, participant level data, and statistical code {31c}

The study protocol and any de-identified database or statistical code required to support the protocol will be supplied on request.

## Oversight and monitoring

### Coordinating center and trial steering committee {5d}

The trial’s day-to-day operations are managed by a coordinating center composed of qualified physicians, study nurses, and clinical scientists. This team is responsible for operational oversight, protocol adherence, and organizational support. A trial steering committee, comprising these same professionals, meets on a monthly basis to review progress and address any issues; meeting frequency can be increased as needed.

### Composition of the data monitoring committee, its role and reporting structure {21a}

An independent Data Monitoring Committee (DMC) is established to oversee participant safety and data integrity. This committee is similarly composed of physicians, study nurses, and clinical scientists ensuring that expertise is maintained across trial oversight functions. The DMC meets monthly, with the meeting interval shortened if required by emerging safety or efficacy concerns.

### Adverse event reporting and harms {22}

Participation may involve certain risks and burdens for the study participants. While the use of high pneumoperitoneum pressure (HPP) aims to reduce intraoperative blood loss and associated complications, there remains a potential risk of CO_2_ embolisms due to the insufflation of carbon dioxide. Additionally, the intraoperative monitoring techniques to detect CO_2_ embolisms, and transesophageal echocardiography, may introduce discomfort or risks typically associated with invasive procedures. Rarely, echocardiography causes arrhythmias, infections, or dental damage. Prolonged use of the echocardiography probe during intraoperative monitoring can result in minor injuries to the mucosal surface and hematomas in the esophagus. Very rarely, esophageal injuries that lead to perforation—especially in patients with preexisting esophageal conditions—can occur and may cause mediastinitis. Participants may also experience anxiety related to undergoing surgical procedures and being part of a clinical trial. Moreover, while efforts will be made to minimize postoperative complications, there is still a possibility of adverse outcomes, such as prolonged recovery or the need for additional interventions. However, it is essential to note that the study protocol has been designed to adhere to strict ethical guidelines, and any risks will be communicated to participants during the informed consent process. Overall, the research team will continuously monitor participant safety throughout the study to mitigate these risks as effectively as possible.

“Adverse events” (AEs) will be recorded on a special AE form in the CRF. AEs will be reported to the principal investigator at regular intervals throughout the study. “Serious adverse events” (SAEs) that result in death, are life threatening, require hospital admission, result in persistent or significant disability or incapacity, or result in reoperation due to any reason will be documented on a special SAEs formula in the CRF and will be reported to the principal investigator within 24 h.

### Frequency and plans for auditing trial conduct {23}

A formal audit of the trial conduct will not be established.

### Plans for communicating important protocol amendments to relevant parties {25}

Important protocol modifications will be reported to investigators, participants, ethics committees, funder, trial registries, and journals.

### Dissemination plans {31a}

Trial results will be published in peer-reviewed journals and disseminated in scientific meetings.

## Discussion

Application of low-pressure pneumoperitoneum in laparoscopic surgery can achieve improvement in postoperative pain scores and facilitate recovery, thereby shortening the length of hospital stay in comparison to standard pressure pneumoperitoneum [23]. Celarier et al. have shown that low-pressure pneumoperitoneum in colorectal surgery can decrease the duration of hospitalization by 1 day in comparison to standard pressure pneumoperitoneum [23]. The results of this prospective randomized trial strengthen the evidence for the application of low-pressure pneumoperitoneum, demanding recommendations for low-pressure pneumoperitoneum to find entrance into the Enhanced Recovery after Surgery protocol [24]. Prospective randomized clinical trials evaluating different pressure pneumoperitoneum in minimally invasive major liver resections are lacking; therefore, evidence-based recommendations on pneumoperitoneum pressure in liver surgery are weak.

Minimizing intraoperative blood loss in MILR is a clinically relevant challenge, greatly impacting the procedure’s safety and influencing the patient’s morbidity and mortality. Positive pressure pneumoperitoneum during MILR is believed to be a main factor regulating intraoperative blood loss through direct vasculature compression [14, 25]. Pneumoperitoneum pressure has been suggested to reduce hemorrhage from hepatic veins in laparoscopic liver resections [26]. For this reason, experts have even suggested applying pneumoperitoneum pressure of 18–20 mmHg to control bleeding during minimally invasive liver resections [27]. Recently, one clinical trial has been published that compares low versus high pneumoperitoneum in MILR [11]. The main objective of this trial was the occurrence of gas embolism. Here, low-pressure pneumoperitoneum reduced the incidence of severe (> grade 2 according to the Schmandra bubble test [28]) CO_2_ embolisms from 67.1% in the standard pressure group to 40.8% in the low-pressure group [11]. Although no significant difference in intraoperative blood loss was established between the two groups, resections adjacent to the second hepatic hilum were identified to specifically benefit from low pressure. We believe that no significant difference in blood loss was detected because 93.6% of surgical procedures were minor liver resections. In contrast, an experimental study on piglets indicated that high intra-abdominal pressure (16 mmHg) does reduce bleeding compared to low-pressure (8 mmHg) pneumoperitoneum [13]. Moreover, CO_2_ embolisms have been shown to occur in this model in 73% of cases but resulted in no significant hemodynamic changes in the majority of animals (94.7%) [29].

In conclusion, high-pressure pneumoperitoneum, exerting counter pressure to the vascular pressure, serves as an efficient tool for bleeding control in MILR. A theoretical risk of CO_2_ embolism, arising from the combination of high intra-abdominal pressure and low central venous pressure, objects the standardized application of HPP. The proposed trial aims to assess non-inferiority of LPP during the parenchymal transection phase of MILR in comparison to balanced HPP.

## Trial status

Recruitment starts in March 2025 and is estimated to be complete by December 2027. The current approved protocol (S-729/2024) is version 1.2 (date of approval: 2024–12–20).

## Supplementary Information

Supplementary Material 1.

## Data Availability

Databases generated by this study will be made available upon reasonable request to the corresponding author.

## Abbreviations

AEs: Adverse events
AS: American Society of Anesthesiologists (score)
BDSG: Bundesdatenschutzgesetz
CO_2_: Carbon dioxide
CI: Confidence interval
CONSORT: Consolidated Standards of Reporting Trials
CRF: Case report form
COPD: Chronic obstructive pulmonary disease
CVP: Central venous pressure
DAMPs: Damage-associated molecular patterns
DMC: Data Monitoring Committee
GDPR: General Data Protection Regulation
HGF: Hepatocyte growth factor
HIF1: Hypoxia-inducible factor 1 (alpha)
HPP: High-pressure pneumoperitoneum
HMGB1: High mobility group box 1
HSP70: Heat shock protein 70
ICC: Intra-class correlation coefficient
ICG: Indocyanine green
IIP: Intraperitoneal insufflation pressure
IL-1: Interleukin-1
IL-6: Interleukin-6
IL-10: Interleukin-10
L-SRS: Leiden Surgical Rating Scale
LPP: Low-pressure pneumoperitoneum
LDSG: Landesdatenschutzgesetz
MILR: Minimally invasive liver resection (or surgery)
mITT: Modified intention-to-treat
MAP: Mean arterial pressure
nDNA: Nuclear deoxyribonucleic acid
mtDNA: Mitochondrial DNA
NYHA: New York Heart Association
PFO: Patent foramen ovale
PDGF: Platelet-derived growth factor
PEEP: Positive end expiratory pressure
Pinsp: Inspiratory pressure
Pmean: Mean airway pressure
POD: Postoperative day
PPULS: Pneumoperitoneum PressUre for parenchymal transection in minimally invasive major Liver Surgery
QoR-40: Quality of Recovery 40
REDCap: Research Electronic Data Capture
rScO_2_: Regional cerebral oxygen saturation
RVOT: Right ventricular outflow tract
SD: Standard deviation
SOPs: Standard operating procedures
SpO_2_: Peripheral oxygen saturation
TNF: Tumor necrosis factor
TOF: Train-of-four
VEGF: Vascular endothelial growth factor
ICU: Intensive care unit
RCI: Reliable change index
NCT: Clinical trial identifier (as used in clinicaltrials.gov)
