# Supplementary material for: High versus low pneumoperitoneum PressUre for parenchymal transection in minimally invasive major liver surgery (PPULS)—a non-inferiority, multicenter, randomized, controlled trial
Source: Trials. 2025 Dec 1;26:556. doi: 10.1186/s13063-025-09269-9 (PMC12670865; doi:10.1186/s13063-025-09269-9)
Supplement: Supplementary file 1 — Supplementary Material 1. [file 13063_2025_9269_MOESM1_ESM.pdf]

UNIVERSITÄTS
KLINIKUM
HEIDELBERG

Klinik für Allgemein-, Viszeral- & Transplantationschirurgie | Universitätsklinikum Heidelberg | Im Neuenheimer Feld 672 | 69120 Heidelberg

High versus low **P**neumoperitoneum **P**ress**U**re for parenchymal transection in minimally invasive major **L**iver **S**urgery
- *A Non-inferiority, Multicenter, Randomized, Controlled Trial*

Consent Form for Patients

..
Name of the Study Participant (in block letters)

Date of birth **...**

I have been thoroughly and comprehensibly informed in a personal conversation by

..
Name of the Doctor or the Informing Person

about the nature, significance, risks, and implications of the study. Furthermore, I have read and understood the text of the information sheet, including both parts. I had the opportunity to discuss the implementation of the study with the informing person and to ask questions. All my questions have been answered satisfactorily.

I had sufficient time to make my decision.

I am aware that I can withdraw my consent to participate in the study at any time, orally or in writing, without giving reasons, and without suffering any disadvantages as a result.

<u>Additional Notes by the Informing Person</u>
I have ensured that the patient was able to ask questions and that the explanation was comprehensible. The following topics or questions were discussed in more detail during the briefing:

Data Protection Consent:

I.

I am aware that personal data about me will be collected, stored, and analyzed in this study. Data processing takes place in accordance with legal regulations and requires the following consent declaration pursuant to Article 6(1)(a) of the General Data Protection Regulation:

I consent to the collection and processing of personal data concerning my health and genetics within this study, both in paper form and on electronic data carriers, as specified in the information sheet Part II.

I agree that health data will be collected from my general practitioner for the purposes of the study. To this extent, I release the respective doctors from their confidentiality obligation.

....
Names of the Doctors

II.

1. I consent to the use of my biological samples as specified in the information sheet Part II.

I agree to be informed about incidental findings:

[ ] Yes
[ ] Yes, provided that there is a likely possibility of prevention or early treatment of diseases
[ ] No

I agree to the extended use of my data beyond the purposes of this study as described in the information sheet:

[ ] Yes
[ ] No

I wish to restrict the use of my data for other/future research purposes as follows:

....

I agree to the extended use of my biological samples beyond the purposes of this study as described in the information sheet:

[ ] Yes
[ ] No

I wish to restrict the use of my biological samples for other/future research purposes as follows:

....

If a hepatitis infection is detected in me, this finding will be reported by name to the responsible health authority. If an HIV infection is detected in me, this finding will be reported anonymously, in an encrypted case-related manner, to the Robert Koch Institute.

I voluntarily consent to participate in the above-mentioned study.

At the same time, I consent to the processing of my personal data and biological samples as described and indicated by me.

I have received a copy of the information sheet and consent form.
A copy remains at the study center.

____________________ ________________________________

Place, Date Name, First Name of Participant (in block letters)

____

Signature of participant

Informing Person

I have informed the participant and obtained their consent.

____________________ ________________________________

Place, Date Name, First Name of Participant (in block letters)

____

Signature of Informing Person
